# A Genome-Wide Association Study Points out the Causal Implication of *SOX9* in the Sex-Reversal Phenotype in XX Pigs

**DOI:** 10.1371/journal.pone.0079882

**Published:** 2013-11-06

**Authors:** Sarah Rousseau, Nathalie Iannuccelli, Marie-José Mercat, Claire Naylies, Jean-Claude Thouly, Bertrand Servin, Denis Milan, Eric Pailhoux, Juliette Riquet

**Affiliations:** 1 INRA, UMR444 LGC, Castanet-Tolosan, France; 2 IFIP, Pôle génétique, la Motte au Vicomte, Le Rheu, France; 3 INRA, UE0332 Domaine de Galles; 4 INRA, UMR1198 BDR, Jouy-en-Josas, France; University of Queensland, Australia

## Abstract

Among farm animals, pigs are known to show XX sex-reversal. In such cases the individuals are genetically female but exhibit a hermaphroditism, or a male phenotype. While the frequency of this congenital disease is quite low (less than 1%), the economic losses are significant for pig breeders. These losses result from sterility, urogenital infections and the carcasses being downgraded because of the risk of boar taint. It has been clearly demonstrated that the *SRY* gene is not involved in most cases of sex-reversal in pigs, and that autosomal recessive mutations remain to be discovered. A whole-genome scan analysis was performed in the French Large-White population to identify candidate genes: 38 families comprising the two non-affected parents and 1 to 11 sex-reversed full-sib piglets were genotyped with the PorcineSNP60 BeadChip. A Transmission Disequilibrium Test revealed a highly significant candidate region on SSC12 (most significant p-value<4.65.10^-10^) containing the *SOX9* gene. *SOX9*, one of the master genes involved in testis differentiation, was sequenced together with one of its main regulatory region Tesco. However, no causal mutations could be identified in either of the two sequenced regions. Further haplotype analyses did not identify a shared homozygous segment between the affected pigs, suggesting either a lack of power due to the SNP properties of the chip, or a second causative locus. Together with information from humans and mice, this study in pigs adds to the field of knowledge, which will lead to characterization of novel molecular mechanisms regulating sexual differentiation and dysregulation in cases of sex reversal.

## Introduction

In mammals, sex differentiation is a genetically and hormonally controlled process. Sex differentiation first relies on the establishment of genetic sex at fertilization by the combination of X or Y chromosome containing gametes to produce XX or XY zygotes. This initial step is then followed by the activation of the appropriate male or female genetic cascades involved in gonad differentiation. In a normal situation, the switch between the male or female pathway is driven by the action of the Y-located testis-determining gene SRY (Sex-determining Region of the Y chromosome) [1]. Once gonad differentiation is engaged, the secretion of testicular anti-Müllerian hormone(AMH) and androgens ensure that sexual characteristics develop correctly into their final form [2]. At any of these steps, flaws in the process can lead to disorders of sexual development. When chromosomal, gonadic and somatic sexes are not consistent, sexual attribution is ambiguous, and both male and female characteristics may be found in a single individual. In mammals, sex reversal has been described in numerous species including humans [3], pigs [4,5], goats [6,7], horses [8], dogs [9,10], mice [11], marsupials [12] and moles [13]. Naturally occurring models of *SRY*-negative XX sex reversal in XX individuals could provide important clues as to the etiology of some cases of the human disorder and increase our knowledge about sex differentiation in vertebrates. These pathologies can result either from loss-of-function of crucial pro-ovarian genes (for example, *FOXL2* in goats [6] or *RSPO1* in humans [14]) or from gain-of-function of a testis-promoting factor (for example, *SRY* or *SOX9* in humans [15,16] or in mice [17,18]). Among farm animals, pigs and goats present a XX intersex phenotype at a higher frequency than other livestock species. These XX sex-reversed individuals have a normal female karyotype (XX) but some degree of testicular differentiation of their gonads [19]. In goats, *SRY*-negative XX sex reversal is due to a recessive mutation, referred to as the "PIS" (Polled Intersex Syndrome) mutation, which is associated with the dominant autosomal trait for hornlessness [6,20]. The causative mutation, identified in 2001, is a large 11.7 kb deletion that affects the transcription of *PISRT1* (PIS-regulated transcript 1) and *FOXL2* (Forkhead box L2). In the homozygous state, this mutation causes the masculinization of all polled genetic females [6]. Despite this successful result in goats, the causal genes in *SRY*-negative XX sex reversal in pigs remain unidentified.

Pig intersex phenotypes range from true hermaphrodites (48%) to male phenotypes with (50%) or without (2%) ambiguities [4]. As in humans some of these cases result either from the presence of XX and XY cells within the same animal (4%) or from the presence of a small fragment of the Y chromosome (containing the *SRY* gene) in XX individuals (2%). However, most XX sex-reversed individuals (94%) do not have *SRY* or any of the other genes normally located on the Y chromosome (*SRY*-negative XX sex reversal). In these cases, testis induction is therefore due, to alterations of autosomal genes in the absence of *SRY* [4]. In pigs, the frequency of XX sex reversal varies from 0.08 to 0.75% depending on the herd, and available heritability estimates for this disorder are high, ranging from 0.72 to 0.81 [21]. Previous segregation analyses with pedigrees of limited size are consistent with autosomal recessive transmission of a single gene [4]. A more recent study however, suggests the possible involvement of multiple autosomal regions [22].

Until recently, the identification of genes causing inherited disorders was performed using genome-wide pedigree-based linkage analyses, or by focusing on candidate genes using population-based association studies. With the recent development of commercial high throughput SNP genotyping BeadChips, these two approaches have merged into a single approach referred to as genome-wide association studies (GWAS). GWAS methods have been shown to be more powerful than linkage based methods [23]. The power of GWAS has been demonstrated by the identification of several genes underlying a range of diseases in humans, dogs and cattle which had been difficult to study previously [24,25].

In this contribution we report the results of a GWAS study of XX intersex in French Large White pigs. We genotyped nuclear families identified by one or several sex reversed offspring using the Illumina PorcineSNP60 BeadChip tool to identify chromosome regions and markers associated with sex reversal. A unique association signal was identified and suggests a probable role for the *SOX9* gene in sex reversal in pigs. The *SOX9* region has been described as a gene desert, presenting extremely conserved elements among mammals. It is now clear that *SOX9* is expressed by means of a complex regulatory region thought to cover at least 1 Mb. Among these sequences, Sekido and Lovell-Badge first located a 3.2-kb testis-specific enhancer of *Sox9* (TES) that was [26] further refined to 1.4 kb and is referred to as the Testis Enhancer Sequence core element (TESCO). Even if *SOX9* was retained as the main candidate gene, TESCO was also candidate for the causative mutation in pigs.

## Materials and Methods

### Animals and Phenotypes

DNA samples were collected from experimental families in the 1990s, and more recently from nuclear families during the national selection program of the French Large-White breed. 

The collection of experimental XX sex-reversed cases was established over a-6 year period starting in 1992 in an experimental herd (Domaine de Galles, Avord, France) of the Institut National de la Recherche Agronomique (INRA). This collection has been described previously [4] and some cases were used for in- depth descriptions of XX sex-reversal, including fetal development [5]. Briefly, all cases (n=30) were tested for the presence of the Y chromosome, especially *SRY*. Then, the parents of true XX sex-reversed cases (94%) were retained in the experimental herd and mated to determine the mode of inheritance of the intersex condition in the pig. The reproductive systems of all intersex cases were examined post-mortem in adults (sporadic cases) or at 5 weeks postnatally (familial cases). As a result, 12 nuclear families comprising 1 to 9 affected piglets and their two non-affected parents were selected.

In addition, samples from 26 half/full-sib families were collected between 2006 and 2010 from the Large-White population of the French breeding companies belonging to the BIOPORC association. In addition to blood sampling, the cases were described by the farmer, the technician or the veterinary surgeon using standard nomenclature. The phenotype was defined using only external anatomical criteria: enlarged vulva, presence of a penile clitoris, number of testicles (0, 1 or 2) located in a scrotum-like structure, and presence of a mid-ventral penile sheath. Animals presenting one or more of these criteria were considered as affected and included in the sample. Samples were also collected from their non-affected parents. These two phenotypic categories, affected and non-affected, were used for association analysis. The animals in the experimental families produced between 1992 and 1998 were described by Pailhoux & al. in the frame of another study [4]. All procedures were approved by the animal welfare commission of the Institute at the time of this study. In our study, only the DNA collected from these animals was used. The second set of samples was collected by the BIOPORC association. The collection of blood samples and external phenotyping of the animals were performed in compliance with the guidelines of the French Ministry of Agriculture and Fisheries.

### Sample genotyping

Genomic DNA was extracted from blood samples using standard protocols. For each sample, the presence/absence of the *SRY* gene was determined by PCR amplification with primer pairs (forward -CTGTAGCCTCTGTGCCTCCT and reverse – TTTCATTGTGTGGTCTCGTG). Amplicons were identified following electrophoresis on agarose gels and samples with amplified products were not retained for SNP genotyping. Additional paternity tests were performed using 10 microsatellites to exclude any families presenting Mendelian inconsistencies from the design. In total, 185 samples were retained, corresponding to 38 Large-White families with in all 89 affected piglets. The samples were genotyped at the Animal Genotyping Platform LABOGENA, (Jouy-en-Josas, France) using the Illumina PorcineSNP60 BeadChip platform (Illumina, San Diego, USA), according to the manufacturer's protocol.

### Quality control and genome-wide association analyses

The analysis was conducted for autosomal SNPs with the PLINK whole genome association analysis toolset (PLINK version 1.07 http://pngu.mgh.harvard.edu/purcell/plink/) [27]. All individuals with a call rate lower than 95% were discarded. Genotype data were obtained for 173 animals (including 89 affected piglets). Markers deviating from the Hardy-Weinberg equilibrium (p<10^-3^) (741 SNPs), markers with a call rate lower than 95% (4944 SNPs), and markers with a minimum allele frequency (MAF) of less than 1% (8804 SNPs) were excluded from the analysis. Using these quality control criteria, 47,155 SNPs out of the initial set of 62,163 SNPs remained available for the Transmission Disequilibrium Tests. Nominal p values corresponding to genome-wide thresholds for significance or suggestive association were determined. We applied a conservative Bonferroni correction [28] considering 47,155 independent tests (i.e. the number of SNPs used), which yielded thresholds of 1.06 × 10^−6^ (significant, log(1/p) = 5.97) and 1.91 × 10^−5^ (suggestive, log(1/p) = 4.72).

### Identification of CNV's

PennCNV software [29] was used to detect pig CNVs surrounding the *SOX9* gene on SSC12. Analysis was performed with all the SNP of the chip (62,163) but only the results obtained on SSC12 were considered. PennCNV software integrates, in a joint-calling algorithm, a Hidden Markov Model (HMM) including the pedigree information when available, the total signal intensity (Log R ratio - LRR) and allelic intensity ratio (B allele frequency - BAF) at each SNP marker, the distance between neighboring SNPs and the population frequency of allele B (PFB). Both LRR and BAF were exported from GenomeStudio (Illumina San Diego, USA) and the PFB file was calculated based on the BAF of each marker. CNV calling was performed using the default parameters of the HMM model with 0.01 as UF factor. The marker positions on SSC12 were derived from the swine genome sequence assembly (10.2) (http://www.ensembl.org/Sus_scrofa/Info/ Index).

### TESCO and *SOX9* sequencing

Five affected pigs, carrying different mutated haplotypes (including the 3 most frequent) were selected for sequencing. These five unrelated individuals were selected from five different nuclear families of the design. In addition, five individuals were selected as controls in families from another project in which no cases of intersexuality were reported. Pairs of primers, covering the entire *SOX9* gene and TESCO regulatory region, were chosen to amplify and sequence these 2 candidate regions (Table S1). The *SOX9* pig sequence is available and annotated in Ensembl (Sscrofa10.2:12:9028879:9033246:1), whereas the TESCO pig sequence was obtained by aligning TESCO human sequence against the pig draft genome (Sscrofa10.2:12:9045199:9047423:1). To sequence PCR products, an aliquot (1–12 µl) was purified in a single step (45 min at 37°C followed by 30 min at 80°C) using 0.5U of Shrimp Alkaline Phosphatase (Promega) and 0.8 U of Exonuclease I (New England Biolabs). Sequencing was performed on a 3730 ABI capillary DNA sequencer using a Big Dye terminator V3.1 cycle sequencing kit (Applied Biosystems, Inc.). Sequences were analyzed using the CodonCode Aligner software v4.0.4 (http://www.codoncode.com/aligner/), and any polymorphisms were detected by comparison with the reference sequence. Multiple alignments of the core region of TESCO was performed with multalin software [30]. In total, five distinct sequences (alleles) were obtained for *SOX9* (Genbank Accession numbers KF422597, KF422598, KF422599, KF422600, KF422601), and 3 for the TESCO region (Genbank Accession numbers KF422602, KF422603, KF422604).

## Results

### Informativeness of the SNP panel

Thirty-eight half/full-sib families comprising 89 sex-reversed piglets were genotyped with the Illumina PorcineSNP60 BeadChip. After quality controls, 173 individuals and 47,155 autosomal SNP were retained for transmission disequilibrium (TDT) analysis. The number of SNPs per chromosome ranged from 1,051 on SSC18 to 5,216 on SSC1. MAFs of polymorphic markers were uniformly distributed between 0.01 and 0.5 (Figure S1). The power of the TDT was directly proportionate to the number of heterozygous parents for markers associated with causative variants, and the level of linkage disequilibrium (LD) between causative variants and SNP markers. The proportion of heterozygous parents for each of the 47,155 SNPs is represented in Figure S2. At least half of the parents of the design were not informative for more than 85% of the SNPs. The LD estimation was computed as reported by Dupuis & al [31] to assess the level of genome coverage provided by the 47,155 remaining SNPs. Each SNP was considered as a pseudocausative polymorphism in order to identify the SNP (and its distance) with the highest r^2^ value (r^2^max) among the remaining markers on the chip located within a 2 Mb surrounding window. Calculations were performed using only the parents of the design. The cumulative frequency distribution of r^2^max is reported in the Figure S3(A). In our pedigree, r^2^max values were lower than 0.5 for 28% of the SNPs and only 25% of the SNPs were in total linkage disequilibrium (r^2^max =1) with another SNP on the chip. The average distance for SNP with a r^2^max of 0.5 was 300 kb (Figure S3(B)). The number of remaining markers and the informativeness of the design indicate that the coverage of the SNP panel may not be optimal for all chromosomal regions.

### Transmission Disequilibrium Test

**Figure 1:**
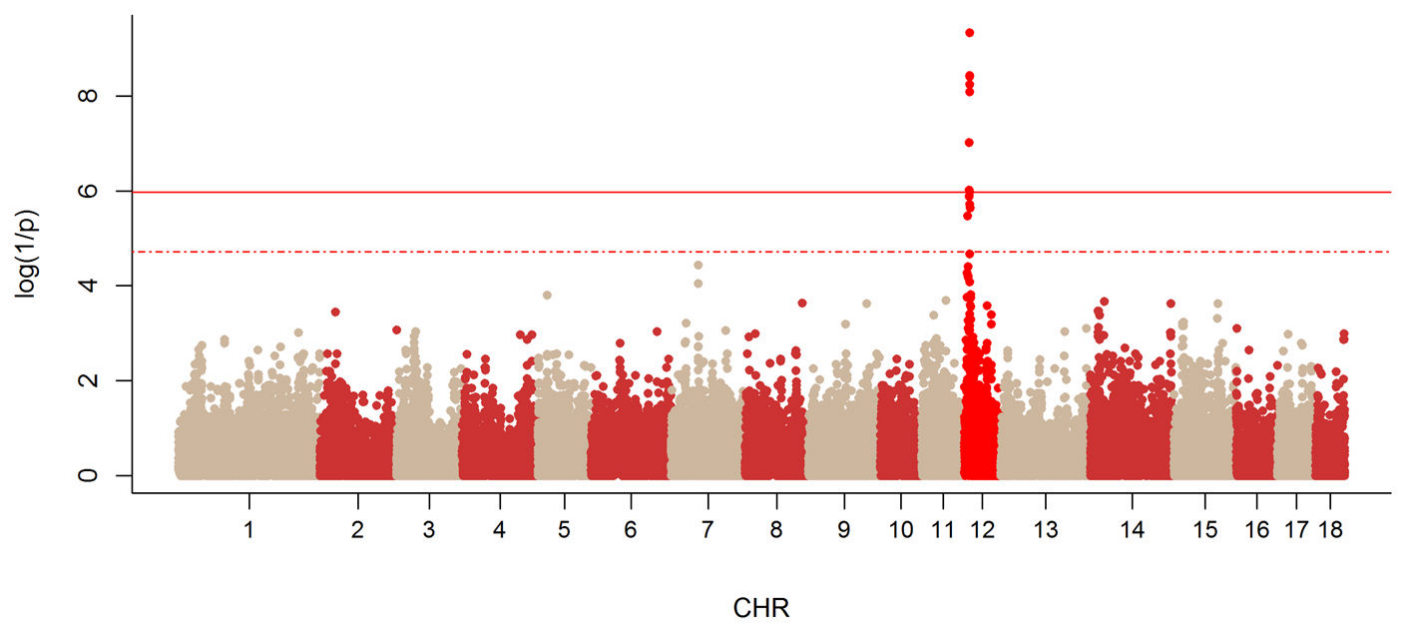
Manhattan plot of genome-wide TDT analysis for sex-reversal phenotype. For each marker the nominal significance (log(1/p)) is indicated. The dotted and the solid lines correspond to the genome-wide suggestive and significant thresholds respectively.

**Figure 2:**
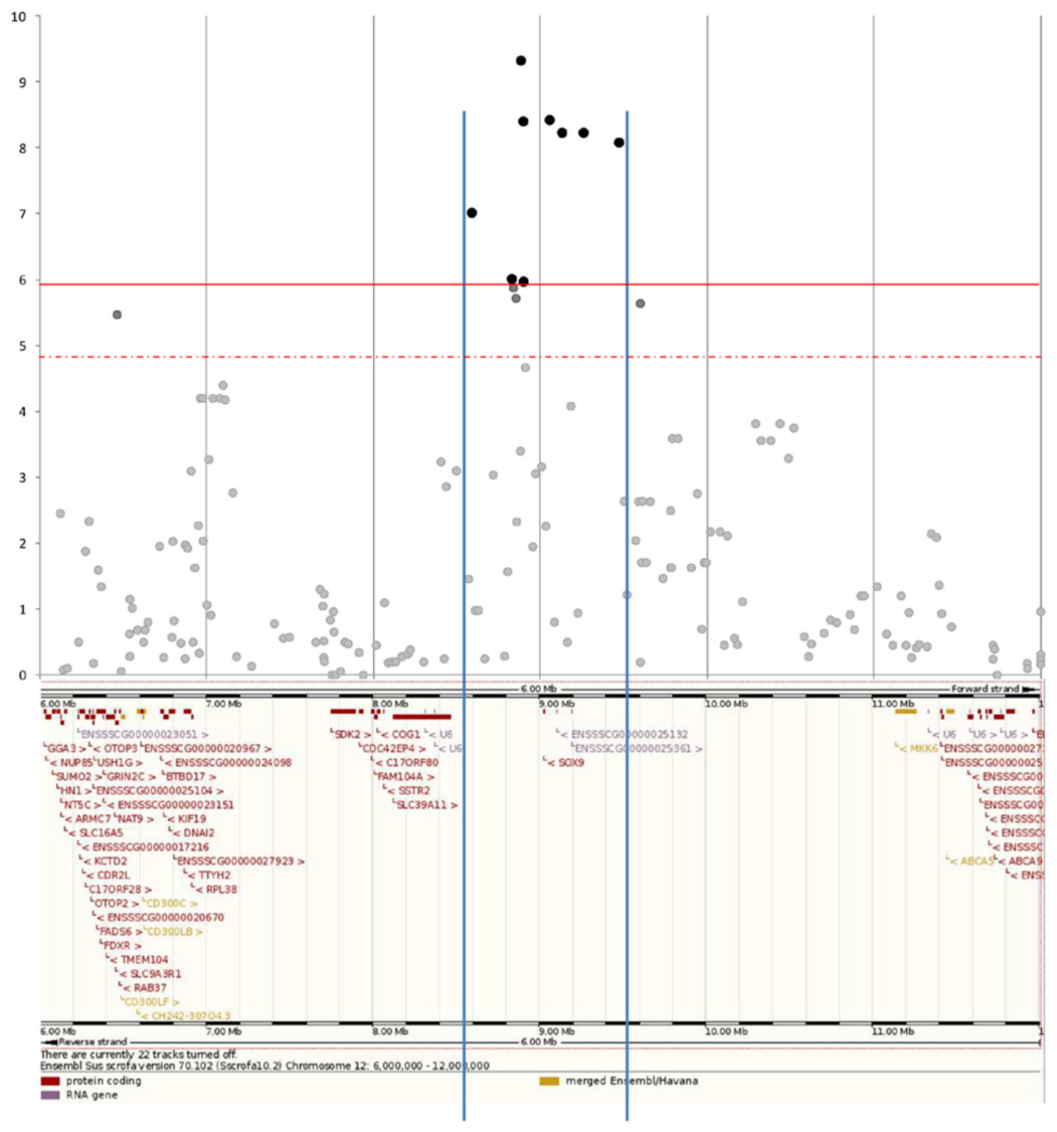
Log(1/p) values of the SNP localized in the 6Mb-12Mb region on SSC12. The non-significant markers are presented in grey, suggestive ones in medium grey, the significant SNP in black. The suggestive (dotted line) and significant (solid line) thresholds are indicated in red. The two vertical lines delimit the minimum interval defined by the first and least significant SNP in the region. At the bottom, genes and transcripts annotated in Ensembl (Sscrofa10.2) are reported.

Due to the two periods of birth of the founders, the population structure was quantified using genome-wide pairwise identity-by-state distances estimated by PLINK. All animals were grouped in a same cluster; these results indicate that despite the differences in the collection periods the two populations have not diverged. The SNPs showing significant genome-wide (p < 1.06 × 10^−6^) and chromosome-wide (1.06 × 10^−6^ < p < 1.33 × 10^−5^) association with the intersexuality (IS) phenotype as determined by TDT are presented in Table 1 and the Manhattan plot in Figure 1. All these SNPs were located on SSC12 between position 6,459,472 and 9,597,291 bp, the most significant marker being located at position 8,881,355 bp (M1GA0024789) (Table 1). Because all these SNPs were localized within the same short interval, it seemed likely that they were linked to a single causal locus. No other significant or suggestive regions were detected by the genome scan. No signals were identified close to PISRT 1 and FOXL2 on chromosome 13, in spite of very informative markers in that region (ALGA0119502 : 39 informative meioses (p=0.53) and ASGA0089628: 43 informative meioses (p=1)). The 3.14 Mb segment encompassing the IS locus on swine chromosome 12 includes several transcriptional units, but only one gene (*SOX9*) was localized in the 882,642 kb segment defined by the position of the significant genome-wide markers (Figure 2). The *SOX9* gene is one of the most important genes downstream from *SRY* in the pathway of testicular differentiation. In the significant interval, *SOX9* was therefore considered as a strong candidate gene.

**Table 1:** Significant SNP association hits in the Transmission Disequilibrium Test for sex-reversal.

**SNP name**	**Chr**	**Position (bp)**	χ^2^	**p-value**	**log(1/p)**
*ASGA0052652*	12	*6 459 472*	*21.6*	*3.36E-06*	*5.47*
ASGA0052953	12	8 586 694	28.47	9.51E-08	7.02
ASGA0098350	12	8 826 005	24	9.63E-07	6.02
*ALGA0112008*	12	*8 835 743*	*23.4*	*1.32E-06*	*5.88*
*ALGA0108362*	12	*8 836 960*	*23.4*	*1.32E-06*	*5.88*
*MARC0011974*	12	*8 851 013*	*22.7*	*1.89E-06*	*5.72*
**M1GA0024789**	**12**	**8 881 355**	**38.82**	**4.65E-10**	**9.33**
MARC0009109	12	8 894 982	34.68	3.89E-09	8.41
ALGA0106073	12	8 897 127	23.82	1.06E-06	5.98
ALGA0064738	12	9 053 320	34.77	3.71E-09	8.43
ASGA0053002	12	9 128 147	33.91	5.78E-09	8.24
H3GA0033370	12	9 257 006	33.91	5.78E-09	8.24
ASGA0097657	12	9 469 336	33.24	8.14E-09	8.09
*MARC0041079*	12	*9 597 291*	*22.35*	*2.27E-06*	*5.64*

Chromosome-wide significant SNP (1.06 × 10−6 < p < 1.33 × 10−5) are in italic, genome-wide significant SNP (p < 1.06 × 10−6) in normal font, and the SNP corresponding to the lowest p-value is highlighted in bold. Positions were defined on the Sscrofa10.2 draft sequence.

### Detection of CNV

To investigate whether a chromosomal rearrangement (deletion or duplication) surrounding the *SOX9* gene could be causal, genotyping data obtained with the 60K SNP chip were analyzed with PennCNV. The analyses of SNP arrays provide normalized total signal intensity and allelic intensity ratios which represent overall copy numbers and allelic contrasts. To detect CNV, we jointly analyzed the 60KSNP genotypes of the 89 affected piglets. Successive windows of 3 markers, corresponding to 150kb windows on average (given the density of the chip) were analyzed. Among all the affected individuals of our design, no structural variations were identified in the 3.14 Mb segment encompassing *SOX9*.

### Haplotypic characterization of *SOX9* surrounding interval

**Figure 3:**
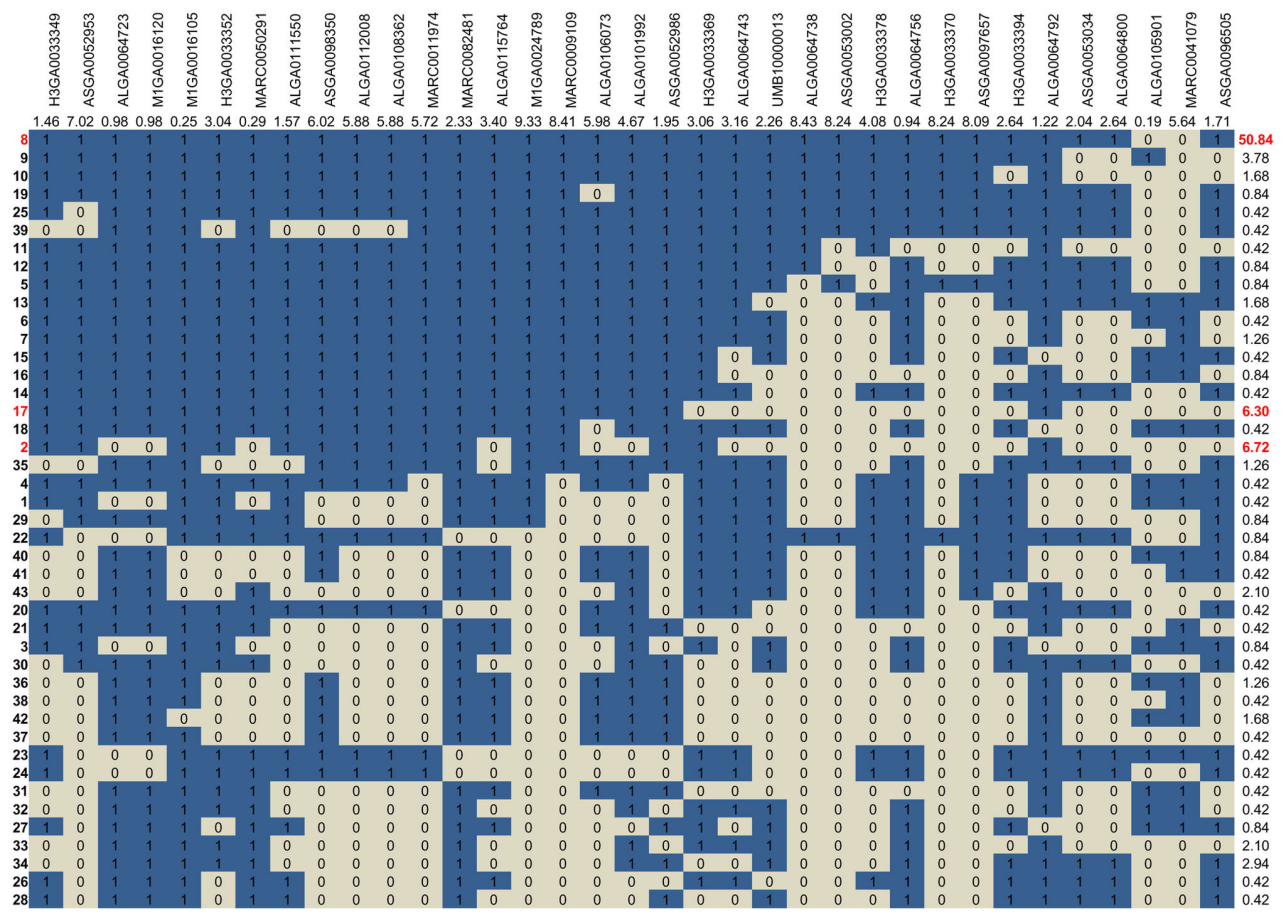
List of the 43 haplotypes defined with the 35 SNP of the chip surrounding *SOX9*. The haplotypes were classified according to the p-value obtained for each marker (sharing of the transmitted allele from the most significant SNP to the less significant one). For each marker the preferentially transmitted allele was noted “1”, the alternative allele “0” and the log(1/p) obtained with the TDT analysis was reported. On the right, the frequency of each haplotype among the affected animals is reported (the 3 most frequent ones are indicated in red). The marker shown in red box is the marker closest to the gene *SOX9*.

To further refine the map position of the causative mutation, 35 SNPs of the chip located between positions 8,566,755 and 9,604,096 bp (interval containing “the significant markers” bounded by an additional marker on each side) were selected to characterize the haplotypes segregating among affected offspring. The phases of each descendant were reconstructed using familial allelic transmission from parents to offspring. Comparing the haplotype-based genotypes, no single homozygous chromosome segment could be identified among the affected piglets of the design. In total 43 different haplotypes segregate within the 38 families. However, among the affected offspring, the frequency of haplotype "8" (50.8%) was much higher than the frequencies of other haplotypes (Figure 3). In addition the frequencies of two other haplotypes, haplotypes 2 (6.7%) and 17 (6.3%) were slightly higher than the others. 

No IBS segment was found in common in the 43 haplotypes, but most of them shared segments of variable size (2 to 30 adjacent markers) identical to the most frequent haplotype. These results suggest that a mutation may be shared by several haplotypes, including the three more frequent ones.

### Candidate region sequencing

The minimum interval mapped by genetic analysis covered 882,642 kb and the causative mutation could be locate anywhere within it. Before undertaking an exhaustive search for all polymorphisms in this region, we chose to target the regions annotated in the interval: the *SOX9* gene and its regulatory region TESCO. We could not exclude that a substitution of an amino-acid residue might increase the lifespan or the DNA affinity of the protein, or block the nuclear exportation signal and lead to abnormal nuclear retention of *SOX9* in the XX gonads [32]. To test whether the causal mutation might be located in the *SOX9* gene or in its regulatory TESCO region, these elements were sequenced in five individuals. Four were homozygous or heterozygous carriers for haplotypes "8", "2" and "17", and the fifth was heterozygous for two rare haplotypes. The aim was (1) to search for candidate mutations that might affect the functional structure of the gene or (2) to identify a small segment shared between the "carrier" haplotypes. Even if a candidate mutation was not identified in the sequences of *SOX9* and TESCO, the presence of a single haplotype shared among all affected individuals would suggest that the mutation is close to these two elements.

**Figure 4:**
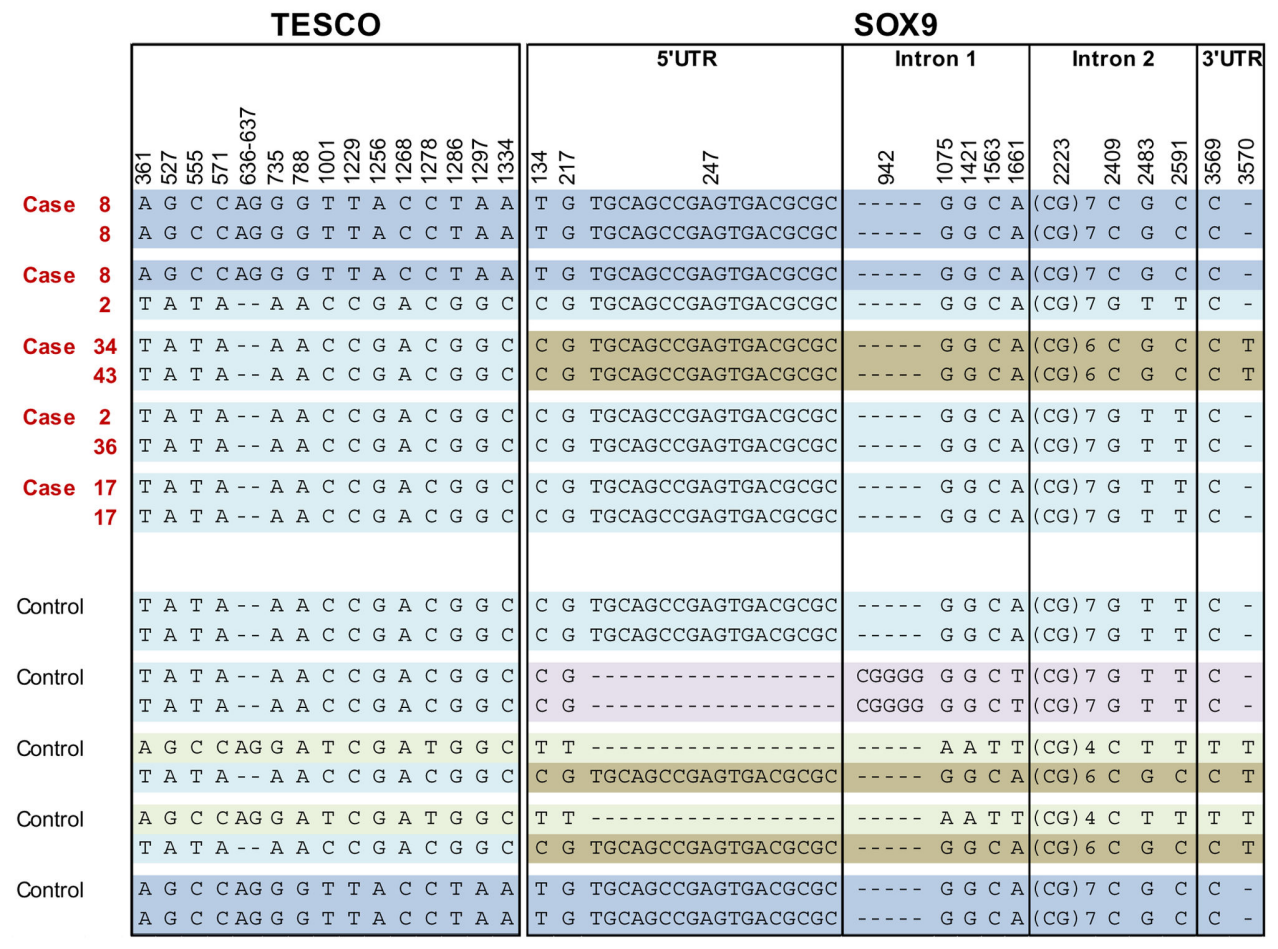
Polymorphisms identified for the 5 cases and 5 controls individuals used for sequencing. For each case, the number in red corresponds to the haplotype number defined with the 35 SNP of the chip surrounding *SOX9*. For each individual the phases defined in *TESCO* and *SOX9* with the detected polymorphisms is reported. Positions were defined from position 9033579 (=1) on the pig draft sequence for *SOX9* and 9047123 (=1) for TESCO. Five different alleles were detected for *SOX9* (Genbank Accession numbers: KF422597, KF422598, KF422599, KF422600, KF422601), and 3 for the TESCO (KF422602, KF422603, KF422604).

Additionally, five healthy animals were also sequenced. The genomic sequence of *SOX9*, including the 5'UTR, the 3 exons, the 2 introns and the 3 'UTR regions, was obtained using 7 pairs of primers and the 1.4kb core region of TESCO was obtained with 4 pairs of primers. In the genomic sequence of *SOX9*, 14 different polymorphisms were identified (10 substitutions and 4 indels from 1 to 18 nucleotides). All these polymorphisms were located outside of the gene exons and outside of the intron / exon junctions. One Indel and 14 substitutions were characterized in the 1.4 kb TESCO region. Consequently, no functional candidate mutation was highlighted (Figure S4). The distribution of the polymorphisms among the sequenced individuals (Figure 4) showed five different alleles for *SOX9* and 3 for TESCO. No shared allele was present between the different "carrier" chromosomes, and identical alleles were identified on "carrier" and "control" chromosomes. Among the control animals, two were homozygous for the same alleles as those characterized in case pigs (Figure S4).

## Discussion

This study focused on the identification of the gene(s) underlying XX sex reversal in the pig. A TDT was conducted using the Illumina PorcineSNP60 array to detect risk loci. In this family-based study, we found genome-wide association with intersexuality for 9 SNPs on SSC12. All these markers are localized in a same small interval that also contains the strong candidate gene, *SOX9*. Some of the experimental families included in the present analysis had been analyzed in 2001 for the expression level of *SOX9* in gonads at 4 developmental stages (50 and 70 dpc; 5 weeks after birth and adult) [5]. This included gonads of XX males with genital ambiguities (mainly testicular tissue) and of true hermaphrodites for which testis-like gonads and ovarian-like gonads had been studied separately. Results had shown that the level of *SOX9* increases in testis-like gonads of XX males and of true hermaphrodites, whereas it remains at a normal female level in the ovarian-like tissues of the latter. However, whereas these results alone were not proof for genetic causality, a *SOX9* genetic determinism has now stronger support with the association signal obtained here. No additional suggestive regions were identified in accordance with a monogenic autosomal recessive mode of inheritance as previously proposed [4].

In the mouse, the transcription factor *SOX9* has been shown to be the direct target of the protein encoded by *SRY* [26]. It has several relevant functions during development and is essential for testis differentiation. In the mouse, *Sox9* is initially expressed in the bi-potential gonad of both sexes at 10.5 day post conception (dpc). It then becomes highly up-regulated in XY gonads and down-regulated in XX gonads at 11.5 dpc [33]. In males, it plays an essential role during Sertoli cell differentiation from supporting cell precursors, which is the preliminary step in testis differentiation [34]. In females, the down-regulation of *SOX9* is maintained in the granulosa cells throughout fetal development. In pigs, *SOX9* is expressed in a similar pattern to that described in mice [35]. Porcine *SOX9* expression during embryonic and fetal development begins at 21 dpc, in both sexes and then increases in the testis from 28dpc on. As in mice, expression in the female is intense as early as 21 dpc, and then decreases from 28 dpc, the key stage of gonadal switch in pigs. 

Both loss- and gain-of-function studies in the mouse support the implication of the *Sox9* gene in gonadal differentiation. A conditional deletion of *Sox9* in the gonads results in XY male-to-female sex reversal [36,37] whereas misexpression of a *Sox9* transgene in XX mice induces female-to-male sex reversal [17,38]. Similar phenotypes have been found in humans. Mutations within and outside of *SOX9* leading to haploinsufficiency are known to cause a bone disorder, campomelic dysplasia, in both sexes and gonadal dysgenesis in 75% of XY individuals presenting as females [39,40]. Even if female-to-male sex reversal in humans is rare, Huang & al. reported in 1999 [16] evidence supporting that *SOX9* duplication, through a *de novo* mosaic 46,XX,dup(17)(q23.1q24.3), can cause XX sex reversal. In 2011, Cox & al described a familial 46, XX Developmental Testicular Disorder due to an approximately 178-kb duplication located 600 kb upstream of *SOX9* [41] and Vetro & al., a 96 kb triplication 500 kb upstream of *SOX9* [42]. Several copy number variants (CNV) and translocations within a large gene desert region upstream of *SOX9* have been characterized as responsible for some of these developmental disorders affecting the skeleton and the genitalia [43,44]. It is now clear that *Sox9* is expressed in various tissues by means of a complex regulatory region thought to cover at least 1 Mb [45]. Based on the large amount of information obtained in other species, we looked for candidate CNVs within the interval detected by TDT that might disrupt regulatory regions. We thus mined our 60K genotyping data for CNVs, but were not able to identify any structural variations. No CNVs have been reported in this interval in previously published studies in different pig breeds [46,47]. Although CNV detection is feasible with this technology, it is impaired by low marker density, irregular distribution of SNPs along chromosomes and the lack of non-polymorphic probes specifically designed for CNV identification. Hence, only the largest CNVs are expected to be detected using the Porcine 60K SNP Chip. We cannot therefore exclude the possibility that CNVs of smaller sizes might exist in the candidate interval. Results obtained in humans with a dense SNP chip reported much higher numbers of CNVs with smaller average sizes than with a less dense SNP chip. Based on our analysis, we cannot exclude that the causal mutation is a small deletion, but it is unlikely that a major structural variation is responsible for the sex-reversed cases studied.

To further refine the localization of the mutation, haplotype analysis was performed on the entire interval. Fourty-three different haplotypes were identified among the affected individuals. Although the hypothesis tested by the TDT analysis was that intersexuality is determined by a single autosomal recessive locus, no shared segment was identified among the different “carrier” haplotypes. However one of the haplotypes (haplotype "8") was shown to be more common than any other, and more than half of the 43 haplotypes had a shared segment of 2 to 30 adjacent markers, with haplotype "8". Although these similarities are consistent with the strong association found in this study, some of the haplotypes identified in sex-reversed individuals were very different from the major haplotype “8”. 

The second targeted analysis that we performed within the candidate interval was the re-sequencing of the *SOX9* gene and TESCO region on different "carrier" and "control" chromosomes. The different elements regulating site- and stage-specific transcription of the *SOX9* gene and all the core regulation elements are still not characterized in detail; however in 2008, Sekido and Lovell-Badge [26] identified a 3.2 kb element responsible for the testis-specific enhancer (TES) of *Sox9*, located 13 kb upstream of the mouse transcription start of the gene. A 1.4 kb core region within TES (TESCO) has been found to be highly conserved across mouse, rat, dog and human genomes , and a 180-bp sequence within TESCO is conserved even in amphibians [48]. No functional candidate mutations were detected within these sequences. The most informative results of this comprehensive analysis of the *SOX9* gene and TESCO variability were (1) the absence of a shared segment between the different "carrier" chromosomes, and to the contrary (2) the presence of identical alleles among "carrier" and "control" chromosomes. Altogether, these results suggest that the causative mutation is not in the sequenced regions, meaning that it is not "in" or "near" the *SOX9* gene or TESCO.

Up to now haplotype analysis and sequencing have not revealed a single haplotype associated with the causal mutation. Some affected individuals carry rare haplotypes very different from haplotype "8". Different hypotheses can be proposed to explain these results: (1) First, these results could be explained if some individuals were incorrectly phenotyped as could be the case when managing collections from numerous breeding farms. Although an information sheet describing the disease was drawn up to ensure that phenotyping was as consistent as possible, we cannot exclude that a few of the "affected animals" were in fact, unaffected. Pig intersex phenotypes range from true hermaphrodites to male phenotypes with or without ambiguities [4]. Most of the sex-reversed animals used in this study were included based on an external morphological description only, and for the TDT all the phenotypic variants were pooled in a single category of affected animals. The wide range of possible phenotypes indicates that the "expressivity" of the trait is variable, and a small number of animals might be poorly phenotyped and mis-classified (2). Secondly the absence of a common segment shared by different "carrier" haplotypes could also be due to the informativeness of the markers used for the analysis. The data obtained so far indicate that none of the SNPs on the chip is in total linkage disequilibrium with the causal mutation. SNP markers on the Illumina PorcineSNP60 BeadChip were selected in order to achieve pan-genetic analyzes, regardless of the breed and whatever the nature of the traits of interest [49]. The LW breed was one of the seven economically important pig breeds used to validate the SNPs selected for the chip, and the highest number of polymorphic loci was obtained for the LW samples. All the families of the XX sex reversal design were from LW breed, which is the best situation (from the breed point of view) to take fully advantage of the informativeness of the markers on the chip. An additional quality criterion used to select the SNPs used on the chip was the MAF value obtained in a validation population panel. The average MAF for the SNPs on the chip is 0.274, and 90% of the SNPs have a MAF >0.15 (criteria of the three first waves of selection). The frequency of XX sex reversed animals in pig populations is less than 1%, and consequently the expected frequency of the mutated allele must be low. It was therefore unlikely that the SNPs on the chip would show strong linkage disequilibrium with the causal mutation. If the chip is of good quality for the analysis of purebred LW animals *a priori*, its use for mapping loci influencing rare trait variations is not necessarily optimal. This sub-optimal situation could explain why no common homozygous haplotype for all the XX sex reversed animals was identified (3). Thirdly, it is necessary to clarify whether allelic (or a genetic) heterogeneity is possible within the analyzed pedigree. The signal obtained on chromosome 12 in the region of *SOX9* targets one mutation affecting gene regulation and certainly explains most cases of XX sex-reversed animals in this design. However, if a second independent gene mutation, also inducing a deregulation of *SOX9*, segregates in the pedigree, some animals could have two mutated copies but be double-heterozygous "affected/+"-"affected/+" with two different haplotypes. In the *SOX9* region, these individuals would be heterozygous for several polymorphisms. Finally, we also cannot exclude that another gene in another chromosomal region might induce an intersex phenotype that is therefore independent of *SOX9*. This hypothesis could not however be confirmed in the present design, as most individuals shared part of haplotype “8”. Further analyses should be conducted by excluding these pigs, leading to a highly reduced number of affected pigs in the residual test population.

So far, the genetic determinism of only a small percentage of sex-reversed pigs is known. The presence and expression of the *SRY* gene (due to chromosomal rearrangement or trisomy) does not however, explain the vast majority of the cases. Here we report the identification of a strong association with genetic markers near the *SOX9* gene. To date, this result has not been reported in pigs and the identification of the causative mutation will add to the knowledge and understanding of sexual differentiation in mammals. The data published here support the continued exploration of the regions that regulate *SOX9* and further mapping of the interval to find the causal mutation. At first, a special effort should be made to increase the power of the design by adding (1) additional individuals and families and (2) SNP markers. Having a larger number of cases would allow help to refine the phenotypes into different sub-categories and understand the variability of the phenotype expression by identifying other potential regulatory genes. In parallel the full re-sequencing of cases and controls in the candidate interval will provide more informative SNP markers presenting a higher LD with the causal mutation. However, the identification of the candidate "8" haplotype means that its frequency can now be estimated in different populations and therefore the number of potentially sex-reversed unexplained cases predicted. From a functional point of view, this haplotype can be used as of now to produce controlled crosses for a detailed exploration of the underlying physiological mechanisms. 

## Supporting Information

Figure S1 **Frequency distributions of minor allele frequencies (MAF).**
(TIF)

Figure S2 **Cumulative frequency of SNP as a function of the heterozygous parents frequency.**
(TIF)

Figure S3 **Estimation of the linkage disequilibrium among the founders of the design.**
(TIF)

Figure S4 **Localization of the detected polymorphisms in TESCO and *SOX9*.**
(TIF)

Table S1 **List of primer pairs used for the amplifications of the *SOX9* and *TESCO* regions.**
(TIF)
