# Supplementary figures and images for: A Genome-Wide Association Study Points out the Causal Implication of SOX9 in the Sex-Reversal Phenotype in XX Pigs
Source: PLoS One. 2013 Nov 6;8(11):e79882. doi: 10.1371/journal.pone.0079882 (PMC3819277; doi:10.1371/journal.pone.0079882)

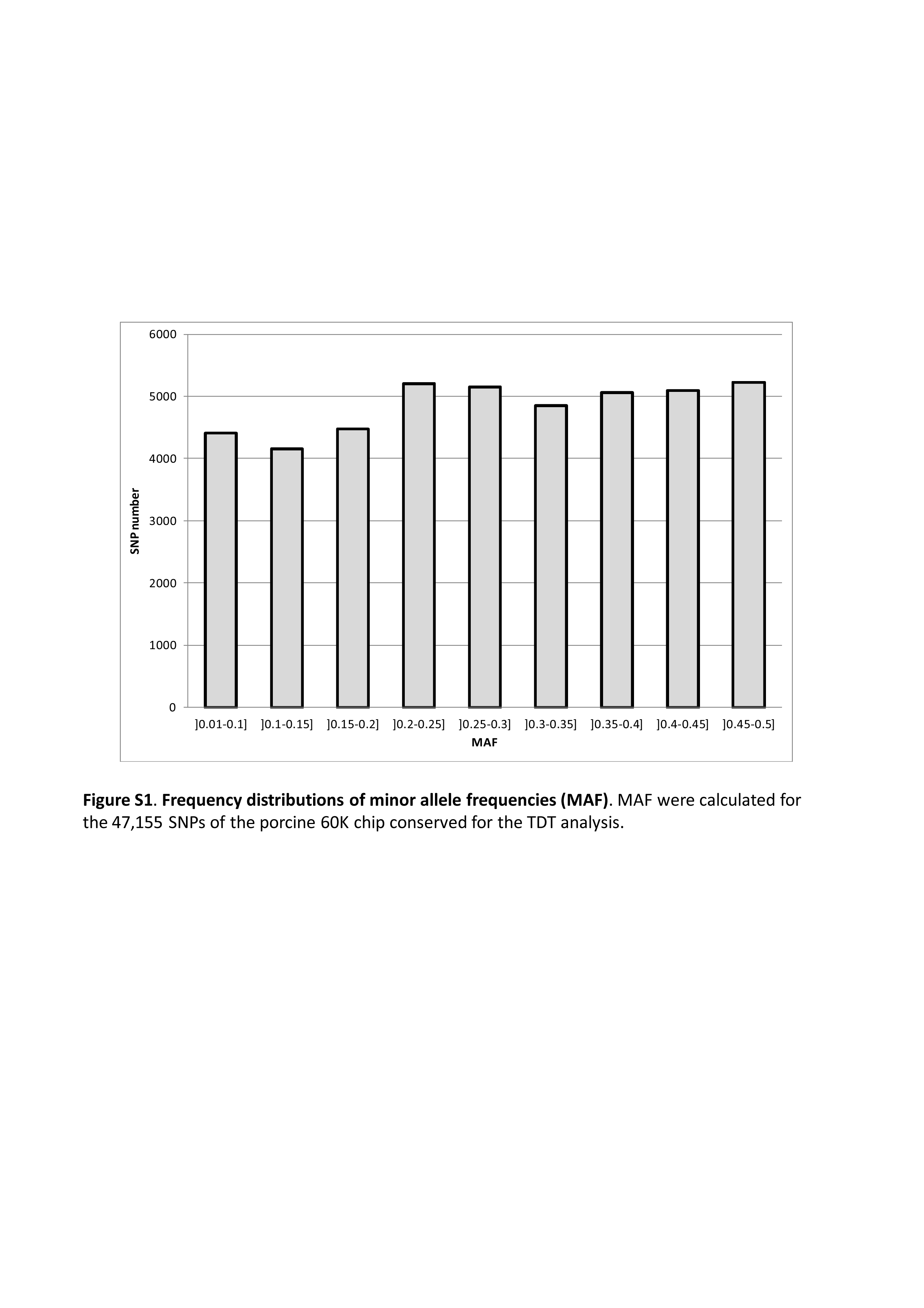

Supplement: Figure S1 — Frequency distributions of minor allele frequencies (MAF). (TIF) [file pone.0079882.s001.tif]

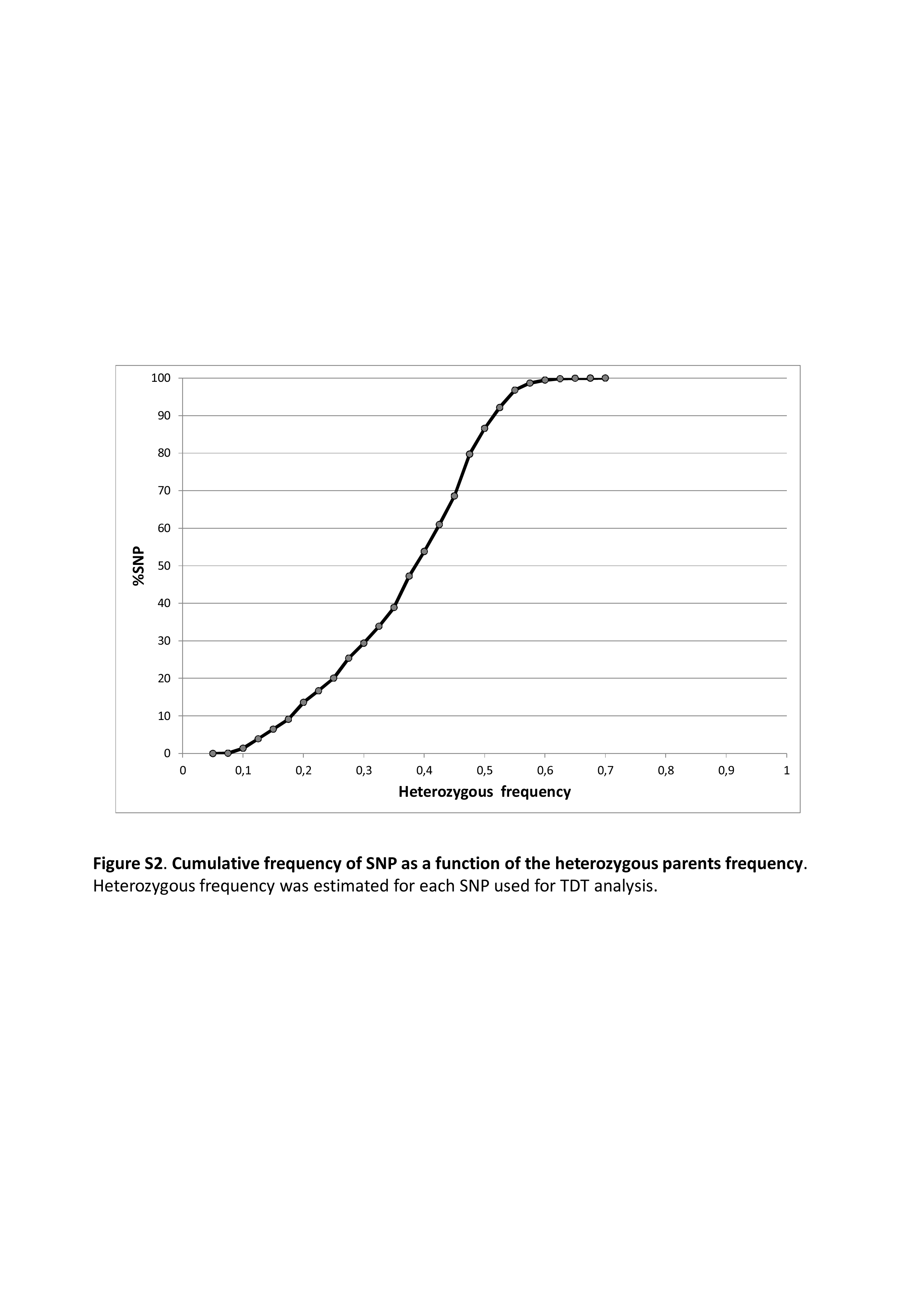

Supplement: Figure S2 — Cumulative frequency of SNP as a function of the heterozygous parents frequency. (TIF) [file pone.0079882.s002.tif]

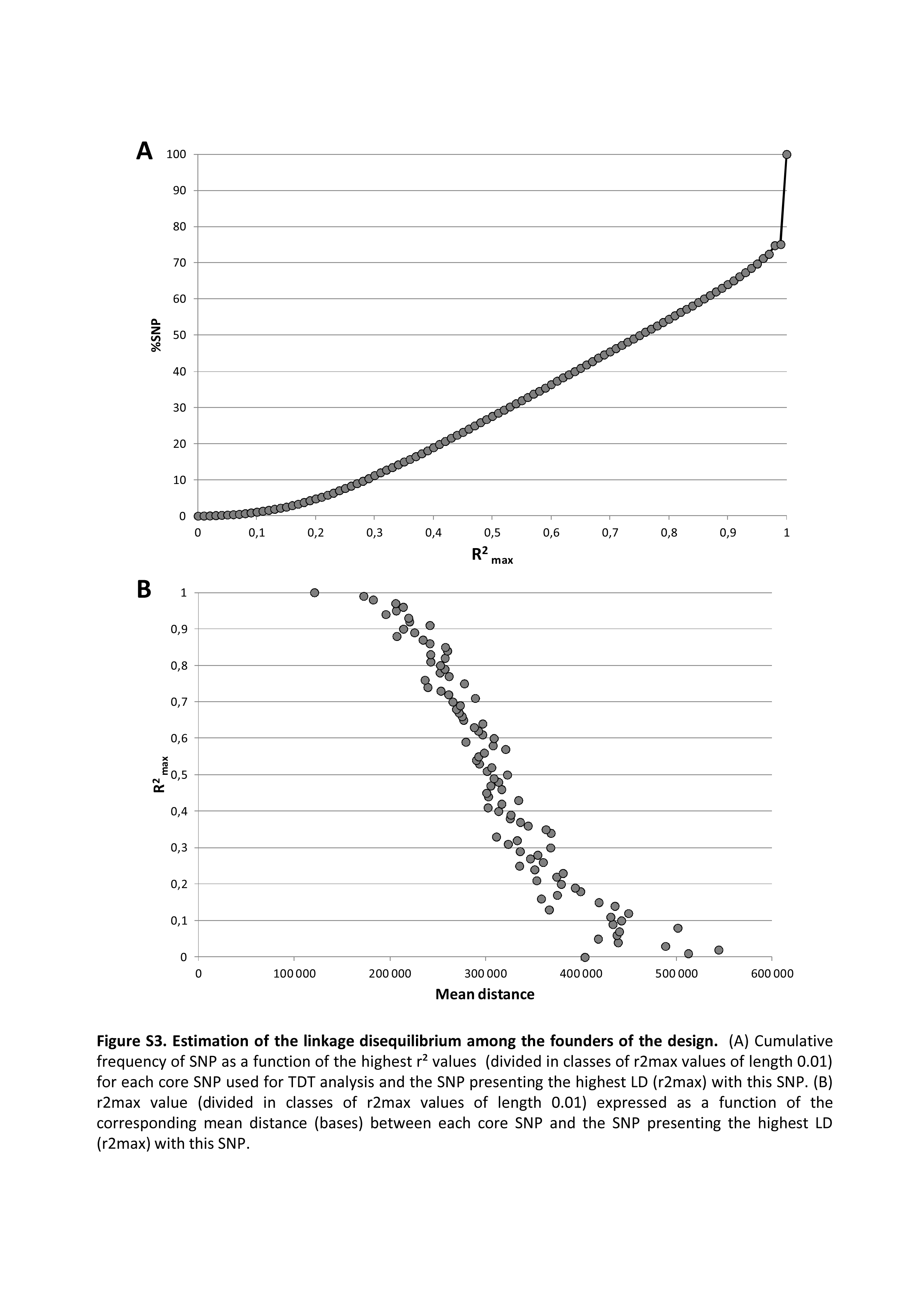

Supplement: Figure S3 — Estimation of the linkage disequilibrium among the founders of the design. (TIF) [file pone.0079882.s003.tif]

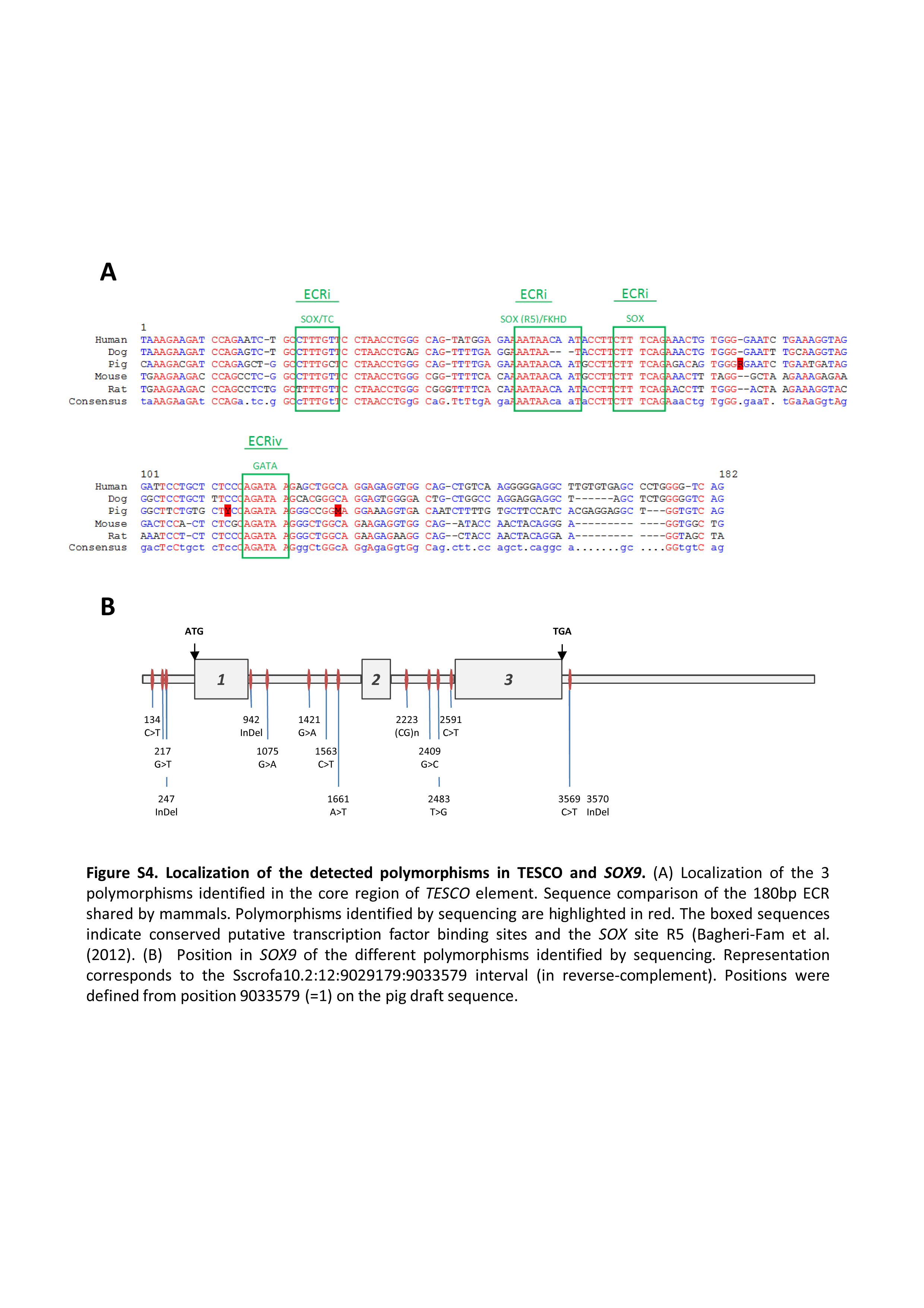

Supplement: Figure S4 — Localization of the detected polymorphisms in TESCO and SOX9. (TIF) [file pone.0079882.s004.tif]

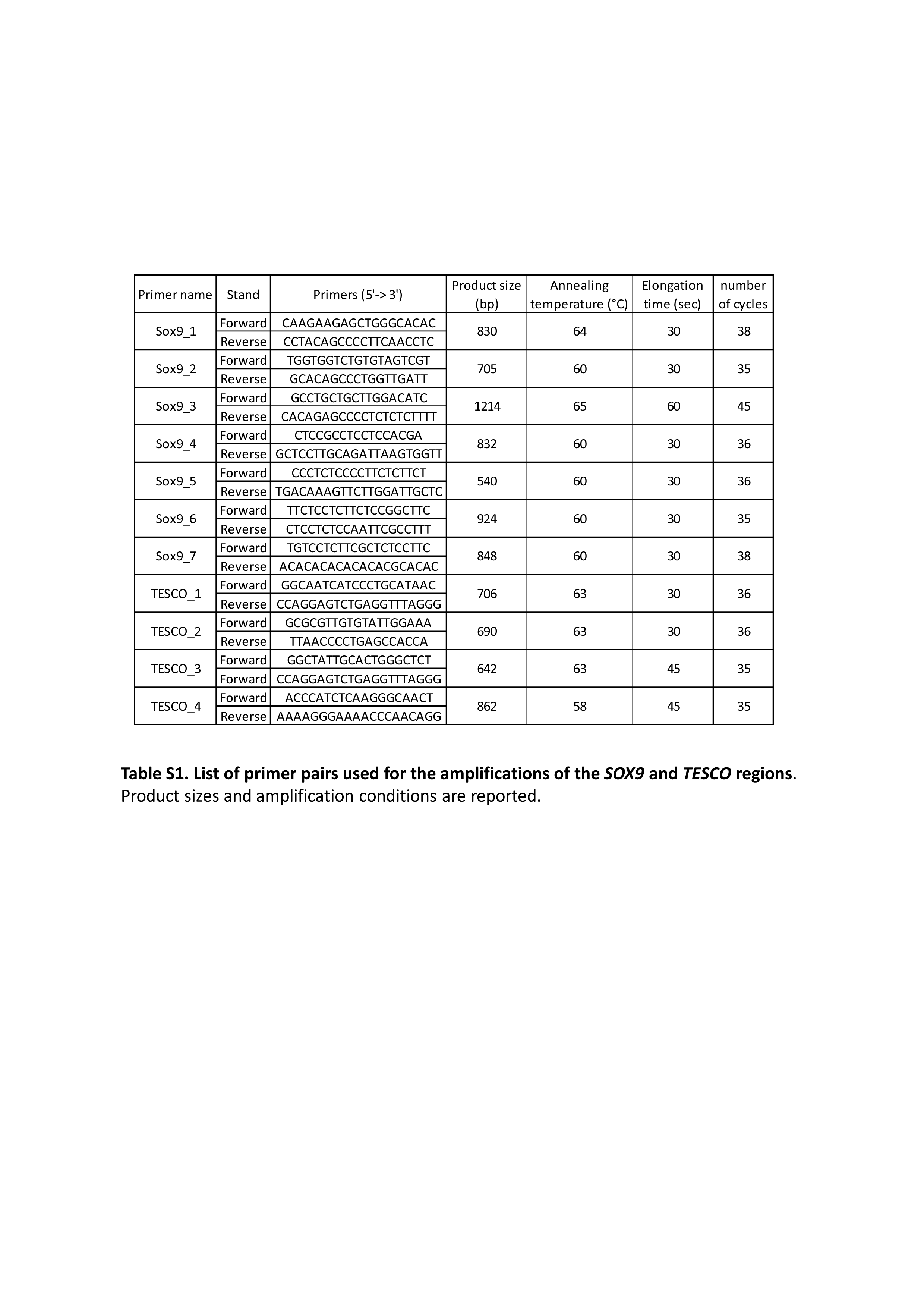

Supplement: Table S1 — List of primer pairs used for the amplifications of the SOX9 and TESCO regions. (TIF) [file pone.0079882.s005.tif]
